# Adaptive, Iterative, Long-Term Personalized Therapy Management in a Case of Stage IV Refractory NSCLC

**DOI:** 10.3390/jpm9030034

**Published:** 2019-07-05

**Authors:** Anantbhushan Ranade, Darshana Patil, Amit Bhatt, Rucha Dhasare, Vineet Datta, Rajan Datar, Dadasaheb Akolkar

**Affiliations:** 1 Avinash Cancer Clinic, Tilak Road, Pantancha Gate, Sadashiv Peth, Pune 411030, Maharashtra, India; 2 Datar Cancer Genetics Ltd., F-8 D-Road, Ambad, Nasik 422010, Maharashtra, India

**Keywords:** non small cell lung cancer (NSCLC), precision oncology, personalized therapy management

## Abstract

In this paper we report long-term therapy management based on iterative de novo molecular and cellular analysis in a case of metastatic non-small cell lung cancer (NSCLC), with prior history of treated colorectal cancer. In the described case temporal tumor evolution, emergent therapy resistance and disease recurrences were addressed via the administration of personalized label- and organ-agnostic treatments based on de novo tumor profiling. This adaptive and iterative treatment strategy countered disease progression at each instance and led to the durable regression of primary as well as metastatic lesions. Concurrently, serial evaluation of mutations in cell-free circulating tumor DNA (ctDNA) via liquid biopsy (LBx) was performed to monitor disease status, ascertain treatment response, identify emergent drug resistance and detect recurrence at sub-radiological levels. The treatment management strategy described herein effectively addressed multiple, sequential clinical conundrums for which viable options were unavailable under the current Standard of Care (SoC).

## 1. Introduction

Standard of Care (SoC) approaches for the diagnosis and treatment of cancers are generally based on parameters such as anatomy, histology and the stage and grade of the disease. In some cancers, the evaluation of selected signaling proteins by immunohistochemistry (IHC) or limited gene variants are additional parameters that guide diagnosis and treatment. In cancers where molecular features are considered for diagnosis or treatment, these are restricted to univariate analyses, which may not sufficiently represent patient-specific cellular and molecular features of the tumor. Likewise, most targeted treatment options under SoC are tethered to the primary organ and are generally based on safety and efficacy data from randomized drug-centric clinical trials. Use of molecularly targeted treatment options in an organ-agnostic setting is encountered less frequently [1,2], with drugs such as Pembrolizumab and Larotrectinib [3] being notable exceptions. 

SoC approaches also do not sufficiently (if at all) factor in the molecular dynamics associated with tumor evolution and drug resistance [4,5]. It then follows that the molecular characterization of the tumor from archival tissue (e.g., from a foundational biopsy) may not be representative of the present status of the malignancy and that treatment choices based on retrospective molecular information may be associated with risks of treatment failure [6]. De novo evaluation of the tumor’s molecular and metabolic dynamics from freshly biopsied tissue or analysis of circulating tumor biomarkers in blood are thus expected to provide the most relevant evidence for treatment selection.

Similarly, SoC approaches towards the monitoring of disease status or response to treatment tends to be based on the manifestation of clinical symptoms of disease or an increase in the severity of symptoms. Though radiological monitoring of disease status is sensitive, it is not viable for high-frequency monitoring due to risks of radiation exposure [7]. Specific serological assays based on tumor antigens are unavailable for many malignancies, and where available may be associated with risks of low accuracy/specificity [8].

Precision Oncology aims to overcome several conundrums associated with SoC approaches in the diagnosis of cancers and treatment selection, by evaluating molecular features of the tumor [9]. The selection of appropriate treatment agents based on molecular evidence and in a label-/organ-agnostic manner is the mainstay of Precision Oncology. There have also been several clinical trials [10,11,12] attempting to match treatments to molecular features of the cancer in an organ-agnostic setting. While some of these trials reported equivocal benefits [10], some others have indicated potential for significant clinical benefits [12] from the administration of molecularly matched therapies. Despite the varying findings of various trials, label-agnostic cancer treatments are often encountered in existing treatment guidelines, e.g., those of the National Comprehensive Cancer Network (NCCN) [13], as well as in routine clinical practice [14] since this can offer treatment avenues for patients where (further) SoC options may be unavailable/unviable. In addition to therapy management, a concurrent aim of Precision Oncology is to improve means for monitoring disease status and treatment response via the evaluation of circulating tumor biomarkers in peripheral blood, viz., liquid biopsy (LBx). LBx interrogates the genomic and metabolic landscape of a tumor by qualitatively and quantitatively evaluating tumor-derived analytes such as cell-free circulating tumor DNA (ctDNA) and exosomal RNAs [15]. LBx can also provide real-time information on tumor dynamics [16] and emergent chemoresistance as well as newer vulnerabilities of the tumor.

Thus, a combinatorial strategy of de novo molecular profiling of cancer along with high-frequency non-invasive LBx monitoring of tumor molecular characteristics can bring an unprecedented level of precision in personalized treatment strategies, especially for advanced, refractory or difficult to treat cancers. In the present report, we describe a case of metastatic non-small cell lung cancer (NSCLC) where long-term therapy management based on de novo real-time evaluation of the multi-layered tumor interactome overcame sequential SoC-therapeutic roadblocks.

## 2. Patient and Methods

### 2.1. Patient

The case described in this manuscript is a retrospective observational report of a single patient who opted to receive personalized cancer treatment. The patient was not part of any prospective interventional clinical trial. The patient provided signed informed consent for the publication of deidentified data and results. Sample collections and therapeutic interventions were carried out at Sanjeevan Hospital, Pune, India and Joshi Hospital, Pune, India. Cellular and molecular investigations on the patient’s samples were carried out at the College of American Pathologists (CAP)-accredited and International Organization for Standardization (ISO)-compliant facilities of Datar Cancer Genetics Limited (DCGL), Nasik, India. As part of standard clinical practices, the patient consented to receive personalized cancer treatment via the treating oncologists at all hospitals where therapy was administered. All interventional procedures including therapy administration were approved as per standard hospital practices and in concordance with existing ethical, medical and legal requirements.

### 2.2. Tissue Collection 

Approximately 5 × 5 × 5 mm freshly biopsied tumor tissue was transferred into 5 mL transport medium (that preserved the viability of tumor cells) and stored at 4 °C during transit. Fresh tissue was either processed immediately or cryopreserved at −80 °C.

### 2.3. Histopathology and Immunohistochemistry

Formalin-Fixed Paraffin-Embedded (FFPE) blocks were prepared as per standard procedures. Histopathological (HPE) and immunohistochemical (IHC) analyses were carried out as per standard procedures. Tumor content of freshly biopsied tissue was determined by HPE evaluations. Tissue samples with ≥80% tumor content were considered as acceptable for molecular evaluations.

### 2.4. Blood Collection and Processing

First, 8–10 mL peripheral blood was collected by venous puncture in each of the Cell-Free DNA BCT^®^ and EDTA vacutainer tubes. Blood was stored and transported at 4 °C. Plasma was separated by centrifugation at 3000× *g* for 20 min at 4 °C, followed by 16,000× *g* for 10 min at 20–25 °C. Plasma from Cell-Free DNA BCT^®^ tubes without hemolysis were processed for cell-free nucleic acid isolation.

### 2.5. Tumor DNA Isolation 

Genomic DNA was isolated from the FFPE tumor blocks using a GeneRead DNA FFPE kit (Qiagen, Germantown, MD, USA) as per the manufacturer’s instructions. DNA was quantified at 260 nm and quality was determined by measuring the ratio of absorbance at 260/280 nm using a NanoDrop 2000 (Thermo Fisher Scientific, Waltham, MA, USA). 

### 2.6. Cell-Free DNA (ctDNA) Isolation

Total ctDNA was purified from 2 mL plasma using a Circulating Nucleic Acid kit (QIAGEN, Germantown, MD, USA) as per the manufacturer’s protocol. ctDNA was quantified using an HS DNA Qubit assay (Life Technologies, Carlsad, CA, USA). 

### 2.7. Tumor RNA Isolation 

Total tumor RNA was isolated using an mirVana miRNA isolation kit (Ambion, Austin, TX, USA) as per the manufacturer’s instruction. Total RNA was quantified using a Qubit 2 Fluorometer (Thermo Fisher Scientific, Waltham, MA, USA) with the manufacturer’s RNA assay kit. 

### 2.8. Exosomal RNA Isolation 

Plasma samples (2 mL) from EDTA tubes were centrifuged at 16,000× *g* for 10 min at 4 °C and filtered via a 0.45 µm membrane to remove larger vesicles. The filtrate was used for the extraction of total exosomal RNA using an ExoRNeasy serum/plasma kit (QIAGEN, Germantown, MD, USA) according to the manufacturer’s protocol [17]. Purified exosomal RNA was quantified using an miRNA Qubit assay (Life Technologies, Carlsad, CA, USA).

### 2.9. Whole-Exome Sequencing (WES)

Tumor genomic DNA was subjected to target enrichment with high multiplex PCR amplification using an Ion Ampliseq^TM^ Exome RDY Kit (Thermo Fisher Scientific, Waltham, MA, USA) and analyzed via an Ion Proton Next Generation Sequencing (NGS) system (Thermo Fisher Scientific, Waltham, MA, USA). DNA reads with a Q17 quality score were aligned to the reference human genome GRCh37/hg19. The average mean depth targeted for the submitted sample was 192× with minimum 100× read depth criteria accepted for variant detection. NGS data were processed using Ion Torrent Suite v5.2 and the Torrent Server was used to successively map the human genome sequence (build GRCh37/hg19) with a Torrent Mapping Alignment Program (TMAP v5.2) optimized for Ion Torrent data. Clinically relevant variants were annotated and classified using American College of Medical Genetics (ACMG) guidelines [18] and variants reported in the literature and databases including HGMD, ClinVar, OMIM, GWAS and COSMIC. Copy number variations were determined with medium sensitivity using Ion Reporter 5.2 software.

### 2.10. Cell-Free tumor DNA (ctDNA) Profiling

A 50-gene NGS panel (Supplementary Table S1) consisting of 207 amplicons and covering over 22,000 bases was designed to detect somatic hotspot mutations reported at high frequency in multiple cancer types as identified from TCGA, COSMIC, ICGC, MD Anderson Cancer Center and My Cancer Genome databases. 

ctDNA (20 ng) was used for NGS library preparation via PCR-based Ampliseq target enrichment protocol. Libraries of 100 pmol were sequenced using Ion Proton (Thermo Fisher Scientific, Waltham, MA, USA). Torrent Suite™ v5.2 (Thermo Fisher Scientific, Waltham, USA) software was used to perform primary analysis, including signal processing and base calling. Primary QC parameters were: minimum read length of 25 bases, read quality trimming of 17 QV, window size for quality trimming 30 bp. The processed sequenced data were aligned to the reference genome GRCh37/hg19 to generate Binary Alignment/Map (BAM) files. Sequencing data were considered for downstream analysis with coverage at ≥10,000× depth and >80% amplicons with at least 600 reads. The aligned data were analyzed using Torrent Variant Caller software with optimized parameters such as minimum allele frequency (0.003), minimum mapping quality (4), minimum coverage (600), down sample to coverage (10,000) and position bias (1). Reported somatic variants of >0.5% allele frequency (AF) were compared to the reference genome hg19. The Integrative Genomics Viewer (IGV) was used to visualize the read alignment and the presence of variants against the reference genome and to confirm the veracity of the variant calls by checking for possible strand biases and sequencing errors. All the germline variants found in the 1000 Genomes Project or The Exome Aggregation Consortium (ExAC) with a frequency of >0.1% were excluded. All somatic mutations were annotated, sorted and interpreted using COSMIC and/or TCGA data. Variants with <0.5% AF were confirmed orthogonally with digital droplet polymerase chain reaction (ddPCR, BioRad) using the rare mutation assay as per the manufacturer’s protocol.

### 2.11. mRNA Profiling (Transcriptome Analysis)

The Ion AmpliSeq™ Transcriptome Human Gene Expression Research Panel was used to determine the expression of 20,802 genes including 18,574 coding genes and 2228 non-coding genes based on University of California Santa Cruz (UCSC) hg19 annotation. Exosomal RNA from asymptomatic individuals (male) was used as a control for cancer exosomal mRNA analysis. RNA prepared from normal tissue was used as a control for tumor mRNA analysis. A barcoded cDNA library was generated with a SuperScript^®^ VILO™ cDNA Synthesis kit from 20 ng of exosomal RNA. The cDNA was amplified using Ion AmpliSeq™ technology as per the manufacturer’s instructions (Thermo Fisher Scientific, Waltham, MA, USA). Amplified cDNA libraries were evaluated for quality on a Bioanalyzer 2100E using a high sensitivity DNA 1000 chip (Agilent Technologies, Santa Clara, CA, USA) and quantified using an Ion Library TaqMan™ Quantitation Kit (Thermo Fischer Scientific, Waltham, MA, USA)/KAPA Library Quantification Kits (KAPA Biosystems/Roche, Basel, Switzerland). Pooled libraries of 100 pM were amplified using emulsion PCR on an Ion Torrent OneTouch2 and enriched as per the manufacturer’s instructions. Templated libraries were sequenced on an Ion Torrent Proton™ sequencing system, using an Ion PI sequencing kit and an Ion PI chip (Thermo Fisher Scientific, Waltham, MA, USA). Analysis of AmpliSeq RNA sequencing data was performed using the AmpliSeq-RNA plugin available for Ion Torrent sequencing platforms. This plugin uses the Torrent Mapping Alignment Program (TMAP—https://github.com/iontorrent/TMAP), which is optimized for aligning raw sequencing reads (from Ion Torrent) against the hg19 transcriptome reference sequence against regions defined in the Browser Extensible Display (BED) file (hg19_AmpliSeq_Transcriptome_21K_v1.bed). The quality of the raw data was evaluated based on three parameters: number of reads, mean read length and target detected (% of all amplicons that had ≥10 assigned reads). Differential gene expression analysis was performed using R/Bioconductor package edgeR with raw read counts from AmpliSeq. Read count normalization was performed using the counts per million (CPM) method. Significant differential expressed genes were called using the following threshold: absolute log fold-change ≥2 and Benjamini–Hochberg adjusted *p* < 0.05. The commercial software iPathway Guide (Advaita) was used for pathway analysis to explore significantly affected pathways.

### 2.12. In Vitro Chemosensitivity Profiling of Viable Tumor Cells

Viable tumor cells were isolated from fresh tissue and treated in vitro with chemotherapy agents and their synergistic combinations. Apoptotic cell death events were determined to evaluate the response to such drug(s). Data from all investigations were integrated to identify agents and their combinations with maximum projected efficacy and safety. 

## 3. Results

This section is divided into four sub-sections, each describing a diagnostic or therapeutic roadblock that was encountered during the management of this case, where SoC approaches may have been unviable or inappropriate. Each sub-section further describes the strategy adopted to overcome the conundrum.

### 3.1. Overcoming Clinical Conundrum #1: Molecular Investigations Facilitated Accurate Diagnosis and Appropriate Therapy Selection 

A 72-year-old never-smoker male patient, known case of diabetes mellitus, was diagnosed in July 2012 with KRAS.pG12D-positive T1N0M0 adenocarcinoma of the ascending colon and cecum. The patient underwent right hemicolectomy with an end to end anastomosis and received oral chemotherapy of Capecitabine (500 mg, Once Daily (OD)) for 4 months. Follow-up (October 2014) 18F-fluorodeoxyglucose (FDG) positron emission tomography-computed tomography (PET-CT) detected hypermetabolic nodular lesion with spiculated margins in the anterior segment of the upper lobe of the left lung, suspected of metastasis from primary Ca colon; the patient received oral chemotherapy of Capecitabine (500 mg, OD) for 2 months. In October 2015, radiological follow-up (chest X-ray) indicated persistent and increased size of lesion in the anterior segment of the left upper lobe of the lung, indicating non-response to Capecitabine. Under SoC treatment strategy, the patient was considered for next systemic treatments for colorectal cancers, which included combinations of 5-fluorouracil, oxaliplatin or irinotecan as well as Bevacizumab.

**Figure 1:**
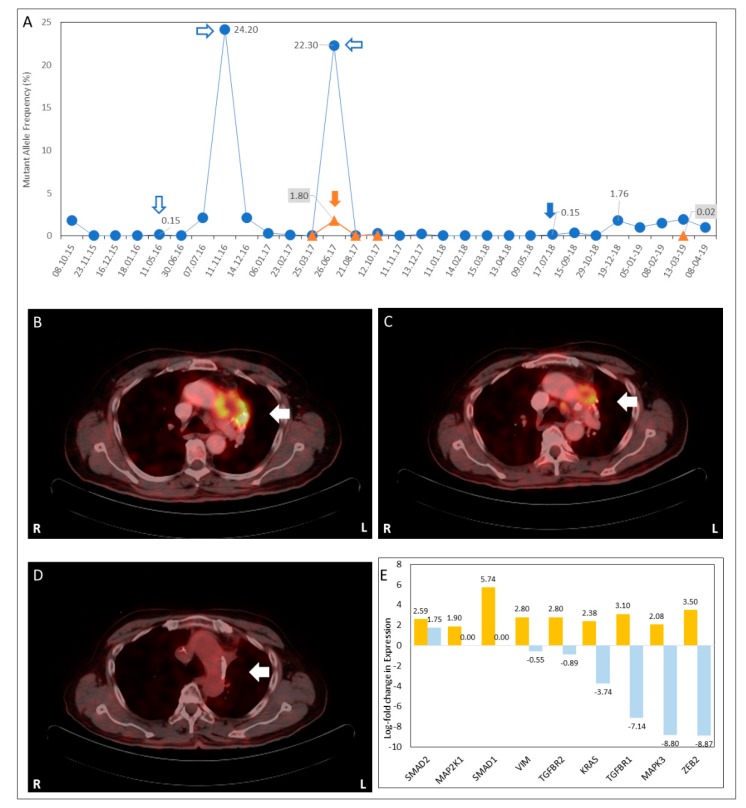
Trends in mutant allele frequency of Epidermal Growth Factor Receptor (EGFR) as determined by liquid biopsy analysis of ctDNA. Variations in allele frequencies of EGFR.pE746-A750del (⏺) and EGFR.T790M (▲) mutations in ctDNA. A spike in EGFR.pE746-A750del (⇩) was observed that was predictive of recurrence. A significant increase in EGFR.pE746-A750del at first recurrence (⇨), second recurrence (⇦) and third recurrence (🡇) was also noted. Detection of EGFR.T790M (🡇) at second recurrence is indicated. By July 2018, the ctDNA EGFR-mutation burden was undetectable (**A**). Treatment response: A regression of 18F-fluorodeoxyglucose (FDG)-avid left prevascular nodal lesion (white arrow) was observed between November 2016 (**B**), January 2017 (**C**) and April 2017 (**D**). L and R indicate left and right sides in the positron emission tomography-computed tomography (PET-CT) transverse sections. Trends in exosomal mRNA profile between November 2016 and January 2017 showed a downregulation of mRNA transcripts, which was suggestive of the reduction in invasiveness and metastatic potential (**E**).

The status of previously reported KRAS as well as other actionable mutations was evaluated via liquid biopsy (LBx) analysis of circulating cell-free tumor DNA (ctDNA) in the patient’s peripheral blood using a commercial multi-gene NGS panel. Interestingly, LBx indicated the absence of the KRAS.pG12D mutation, but the presence of an exon 19 deletion mutation (pE746-A750del) in the *Epidermal Growth Factor Receptor* (*EGFR*) gene. This mutation was previously reported in non-small cell lung cancers (NSCLC) and indicated potential benefit from EGFR tyrosine kinase inhibitor (TKI) therapies [19]. In view of this molecular evidence, the lung mass was biopsied and evaluated by histopathological examination (HPE), which indicated that the tumor was adenosquamous (ADS) subtype, positive for CK7, P63, CK5/6 and CEA, but negative for TTF1. In view of molecular and HPE evidence, the diagnosis was confirmed as a second primary of NSCLC. Based on the sensitizing EGFR mutation, the patient was assigned a regimen of Gefitinib (tablet, 250 mg, OD) for 3 months. In January 2016, the patient underwent upper lobectomy and mediastinal lymph node dissection through a posterolateral thoracotomy, and later continued to receive Gefitinib therapy. High-frequency serial monitoring of EGFR exon-19 mutation burden in ctDNA indicated decreasing mutant allele frequency (MAF) (Figure 1A) concurrent with therapy response.

### 3.2. Overcoming Clinical Conundrum #2: Monitoring for Sub-Radiological Disease and Recurrence

SoC approaches to monitoring of disease status and treatment response are based on the manifestation of clinical symptoms, in absence of which the disease status remains unknown. In the present case, disease status was monitored at high frequency via LBx evaluation of mutation burden in ctDNA while the patient was on Gefitinib therapy. In May 2016, follow-up LBx detected a transient spike (Figure 1A) in EGFR exon-19 MAF, though the patient was clinically asymptomatic. At the next scheduled PET-CT scan (July 2016) there was no radiological evidence of recurrence or progression. The patient continued to receive Gefitinib therapy. Subsequently, EGFR exon-19 MAF was undetectable by LBx (June–October 2016) until November 2016, when a significant increase (Figure 1A) in EGFR exon-19 MAF was detected by LBx. Based on this observation, PET-CT scan was performed which showed equally significant increase in size and metabolic activity (maximum specific uptake value, SUV_Max_) of the left lung upper lobe mass lesion, which contiguously infiltrated into the mediastinum. An additional FDG-avid (metastatic) lesion was detected in the left adrenal gland. In the present instance, the detection of the transient spike appeared to be indicative of disease recurrence at sub-radiological levels and prompted close monitoring of the patient, which led to timely detection and radiological confirmation of disease recurrence.

### 3.3. Overcoming Clinical Conundrum #3: Personalized Treatment Selection when Viable SoC Treatment Options Were Unavailable 

At recurrence, surgical resection of the lung lesion as well as irradiation therapy were deemed unviable owing to the size and location of the lesions. HPE of freshly biopsied tumor tissue from the lung lesion indicated squamous-cell (SCC) morphology and appeared to be suggestive of a histopathological shift. Analysis of mutations in ctDNA indicated significant EGFR exon-19 MAF. However, whole-exome sequencing (WES) analysis of DNA obtained from SCC tissue was unable to detect EGFR exon-19 deletion mutation, suggesting that SCC tissue was a consequence of discrete clonal evolution. Thus, the patient appeared to harbor at least two subtypes of the malignancy based on EGFR mutation, one being the EGFR-positive ADS and the other being the EGFR-negative adenocarcinoma (ADC). However, since the adrenal lesion was unsuitable for biopsy and due to the inability to radiologically identify other metastatic sites, the simultaneous co-existence of histopathologically heterogeneous tumor subtypes could not be ascertained.

SCC subtypes of NSCLC are generally associated with poorer prognosis and an absence of viable treatment options [20]. Actionable molecular indications are also generally unknown in SCC. In order to address this therapeutic roadblock, comprehensive evaluation of circulating tumor biomarkers in peripheral blood was carried out, which identified multiple potentially targetable features such as the overexpression of *EGFR* and *ERBB2* genes as well as the upregulation of pathways such as *MAPK/ERK* pathway (genes including *KRAS, MAP2K1, MAPK3*) and Epithelial to Mesenchymal Transition (*EMT*) pathway (genes including *MMPs TGFBR1, TGFBR2, ZEB, SMAD* and *Vim*).

A combinatorial therapeutic strategy was designed to achieve: (a) the inhibition of *MMPs* via Doxycycline [21,22], (b) the suppression of EMT via Atorvastatin [23,24], (c) the perturbation of microtubule dynamics by Paclitaxel and (d) the targeting of *EGFR* as well as *ERBB2* with Afatinib.

The combination of Afatinib and Paclitaxel has been reported to be beneficial in Gefitinib/Erlotinib-resistant NSCLC with upregulated *ERBB*-family signaling receptors [25,26]. The anti-tumor activity of these drugs (single agents) as well as their combinations were determined by in vitro chemosensitivity analysis using viable tumor cells obtained by fresh tissue biopsy. Based on these findings, the patient was assigned (November 2016) a regimen of Afatinib (40 mg, OD), Paclitaxel (80 mg/m^2^, weekly), Atorvastatin (20 mg, 1 Thrice Daily (TD)) and Doxycycline (100 mg, 1 Twice Daily (Bis Daily, BD)).

Follow-up (January 2017) LBx, while the patient was receiving therapy, indicated (Figure 1A) a concomitant decrease in ctDNA *EGFR* mutation load as well as the downregulation of transcripts associated with *EMT* (Figure 1B) and *MAPK/ERK* pathways (Figure 1B), indicating reduced potential for invasion and metastasis. Supplementary Figure S1A–D depicts the changes in pathways and regulatory associations between pathway intermediates associated with *EMT* and *MAPK/ERK* over the period of November 2016 to January 2017. Simultaneously, follow-up PET-CT scan showed a regression of prevascular mass (Figure 1C) and adrenal lesions. Radiological regression of the metastatic lesions rendered them amenable to CyberKnife^®^ radiosurgery; a dose of 48 Gy was administered in eight fractions (January–February 2017) to the prevascular lesion and pretracheal node, followed by 12 Gy across two fractions to the prevascular lesion and 48 Gy across three fractions to the adrenal gland. Radiosurgery was followed by a continuation of combination therapy. Further reduction in ctDNA EGFR exon-19 MAF (March 2017) was accompanied by near complete radiological resolution of all previously noted lesions, the resolution of pericardial effusion and the absence of new lesions.

In absence of SoC treatment options, the clinicians availed of evidence-based label-agnostic treatment options for the patient which not only led to disease regression, but also to the re-establishment of the viability of an established loco-regional SoC treatment option.

### 3.4. Overcoming Clinical Conundrum #4: Combination of Agents Addresses EGFR Resistance and Target Latent Vulnerability of Tumor

Follow-up (June 2017) LBx evaluation of EGFR mutations in ctDNA detected EGFR.pT790M [27] mutation in ctDNA. The subsequent PET-CT (July 2017) showed radiological evidence of disease progression with new metastatic lesion in the liver and suspicious FDG-avid focus in pericardium. Biopsy of metastatic tissue from the liver and HPE analysis indicated that the tumor cells were adenocarcinoma—a second histopathological shift which indicated the presence of multiple histopathological subtypes. Immunohistochemistry (IHC) analysis was indicative of Androgen Receptor (AR) overexpression. Recent studies have indicated the therapeutic relevance of targeting AR where it is overexpressed in malignancies other than that of the prostate [28]. The patient was assigned a combination therapy with the third generation EGFR-TKI Osimertinib (80 mg, 1 OD) and the AR-antagonist Bicalutamide (50 mg, 1 OD), which led to steady regression of metastatic lesions (June 2017–August 2018) along with a concomitant decrease in ctDNA mutation burden. 

### 3.5. Recent Status

**Figure 2:**
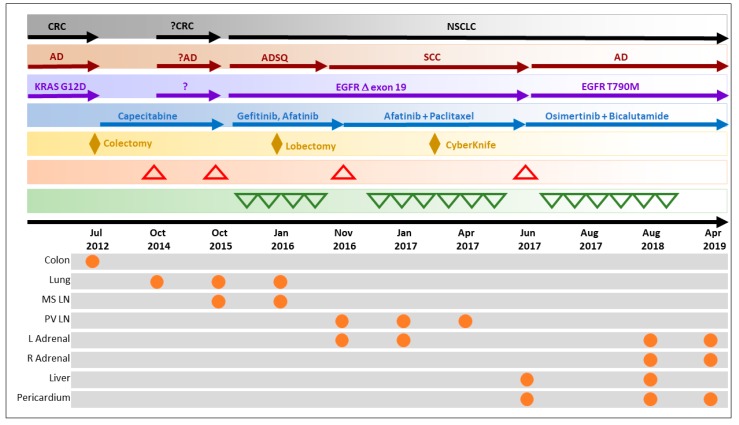
Timeline of events. CRC: colorectal cancer; NSCLC: non-small cell lung cancer; AD: adenocarcinoma; ADSQ: adenosquamous carcinoma; SCC: squamous cell carcinoma; ⬧: locoregional treatment; **△**: progression/recurrence; **▽**: treatment response/regression; ⏺ presence of malignant mass at various sites; MS LN: mediastinal lymph node; PV LN: prevascular lymph node; L Adrenal: left adrenal; R Adrenal: right adrenal.

In the end of July 2018, follow-up LBx indicated a marginal spike in EGFR exon-19 deletion MAF (Figure 1A). Follow-up PET-CT (Aug 2018) indicated a decrease in the size and extent of liver lesions and a stable size of the pericardial lesion, but the appearance of new FDG-avid focus in the right adrenal gland, which was not amenable to biopsy. Due to the deterioration of the Eastern Cooperative Oncology Group (ECOG) performance score, the patient was considered unfit for aggressive treatment regimens. Combination treatment with Bicalutamide and Osimertinib was paused between September 2018 and February 2019, during which ctDNA EGFR exon-19 deletion MAF was observed to increase. Follow-up PET-CT in December 2018 did not report liver lesions, but indicated interval increase in the size of FDG-avid foci in the right adrenal gland and pericardium. In February 2019, the patient resumed a regimen of Osimertinib + Bicalutamide, which led to a decrease in EGFR MAF between February and April 2019. However, due to significant systemic deterioration, the patient was taken off therapy in April 2019. The patient subsequently passed away in April 2019 following terminal cardiorespiratory arrest possibly due to long-term exposure to Osimeritinib [29]. The sequence of events is summarized in Figure 2.

## 4. Discussion

Discrete clonal evolution under the selection pressure exerted by the targeted molecules pose a significant challenge in controlling the disease at the recurrence stage. In this patient, at the first recurrence, existence of discreet EGFR-negative and -positive clonal sub-populations were identified by comprehensive tissue- and blood-based analyses. Such populations could have responded differently if the single drug approach would have followed. The combined use of Paclitaxel and Afatinib effectively controlled the disease at all the locations. The liquid biopsy analysis confirmed treatment response by demonstrating a decrease in *EGFR* mutation load and the downregulation of EMT markers. At the second recurrence, the reappearance of an *EGFR*-positive population in the liver lesions with selective evolution to T790M further enabled the successful use of Osimertinib. Approximately 30–70% of NSCLC cases have AR expression [28]. As AR signaling has been shown to be intact in such patients, AR blockade could be a potential endocrine treatment. In the present case, the combination of Osimertinib and Bicalutamide was well tolerated at a standard dose, with no instance of dose-limiting toxicity, and demonstrated clinical efficacy in controlling the multiple clonal population. 

The adaptive, iterative evidence-based treatment approach that guided treatment selection helped overcome successive therapeutic challenges, avoided unfavorable outcomes and unambiguously contributed to life extension for the patient.

It is pertinent to state that during the timeline of events (2012–2019) described in this manuscript, Osimertinib was first approved by the United States Food and Drug Administration (USFDA) in 2015 for use in non-small cell lung cancers (NSCLC) harboring mutations in exon 20 (T790M). However, it was only in April 2018 [30] that the USFDA approved Osimertinib for use as front-line treatment for NSCLC harboring *EGFR* mutations targetable via the TKI class of drugs.

Dosages of *EGFR* TKIs Gefitinib, Afatinib and Osimertinib were based on labeled indication in lung cancer. The dosage of Paclitaxel (when given in combination with Afatinib) was based on safety and efficacy data reported in the original trial. The dosages of Bicalutamide, Doxycycline and Atorvastatin were based on safety data from the drug labels. All drug dosages were finalized by the treating oncologists based on the patient’s fitness and risk of adverse events.

## Data Availability

Deidentified data may be made available by the authors upon reasonable request, and may require the execution of appropriate non-disclosure agreements.

## Supplementary Materials

The following are available online at https://www.mdpi.com/2075-4426/9/3/34/s1, Figure S1: Molecular pathways linked to invasion and metastasis. TGF-β signaling pathway intermediates in November 2016 (A) and January 2017 (B). ERBB2 and MAPK/ERK pathway intermediates in November 2016 (C) and January 2017 (D), Table S1: List of genes in multigene NGS panel.
