# Supplementary material for: Adaptive, Iterative, Long-Term Personalized Therapy Management in a Case of Stage IV Refractory NSCLC
Source: J Pers Med. 2019 Jul 5;9(3):34. doi: 10.3390/jpm9030034 (PMC6789881; doi:10.3390/jpm9030034)
Supplement: Supplementary file 1 [file jpm-09-00034-s001.zip › Supplementary Table 1.docx]

Supplementary Table 1. List of genes in multigene NGS panel.

| ABL1 | EGFR | GNAQ | KRAS | PTPN11 |
| --- | --- | --- | --- | --- |
| AKT1 | ERBB2 | GNAS | MET | RB1 |
| ALK | ERBB4 | HNF1A | MLH1 | RET |
| APC | EZH2 | HRAS | MPL | SMAD4 |
| ATM | FBXW7 | IDH1 | NOTCH1 | SMARCB1 |
| BRAF | FGFR1 | IDH2 | NPM1 | SMO |
| CDH1 | FGFR2 | JAK2 | NRAS | SRC |
| CDKN2A | FGFR3 | JAK3 | PDGFRA | STK11 |
| CSF1R | FLT3 | KDR | PIK3CA | TP53 |
| CTNNB1 | GNA11 | KIT | PTEN | VHL |
