# Supplementary figures and images for: Adaptive, Iterative, Long-Term Personalized Therapy Management in a Case of Stage IV Refractory NSCLC
Source: J Pers Med. 2019 Jul 5;9(3):34. doi: 10.3390/jpm9030034 (PMC6789881; doi:10.3390/jpm9030034)

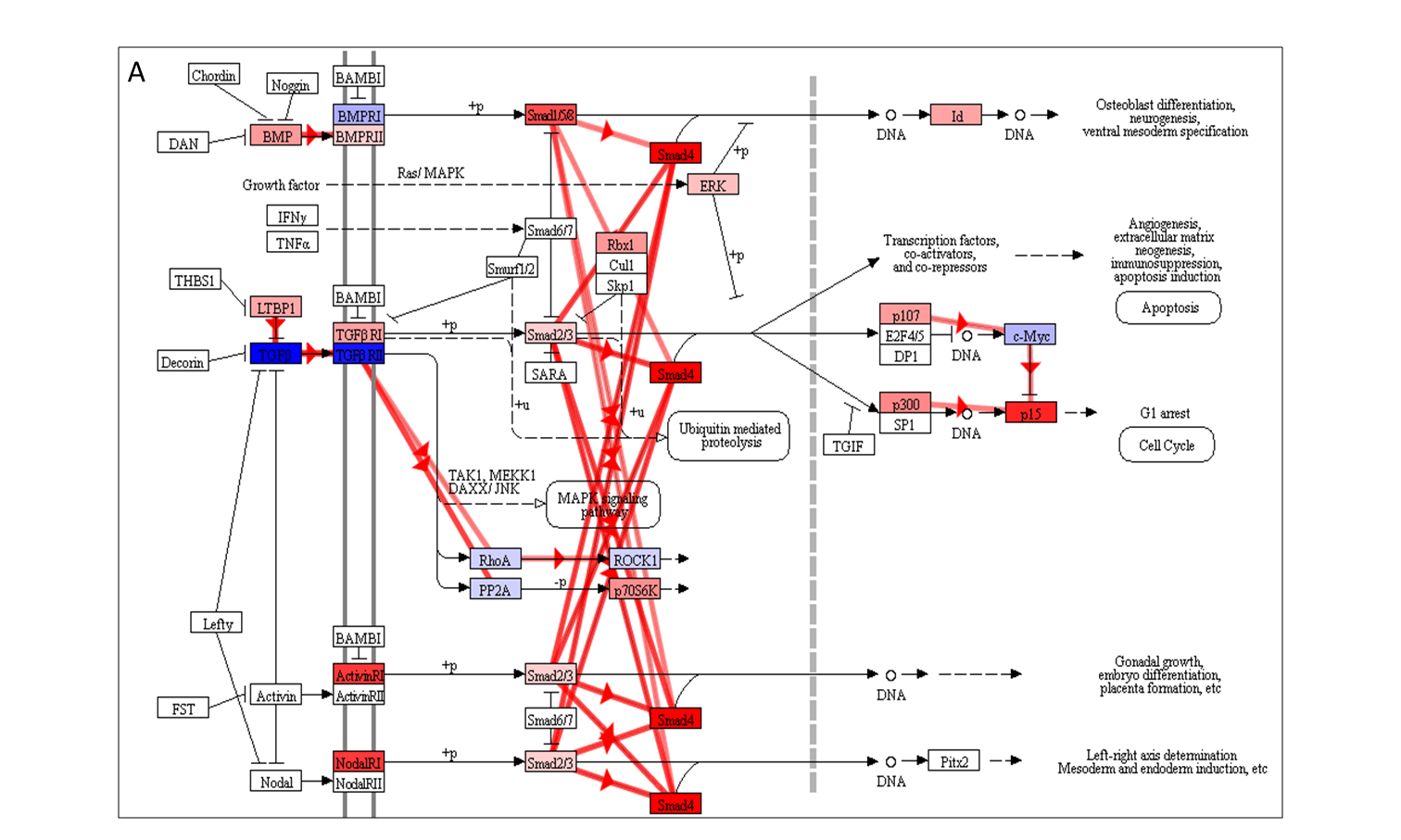

Supplement: Supplementary file 1 [file jpm-09-00034-s001.zip › Supplementary Figure S1A.TIF]

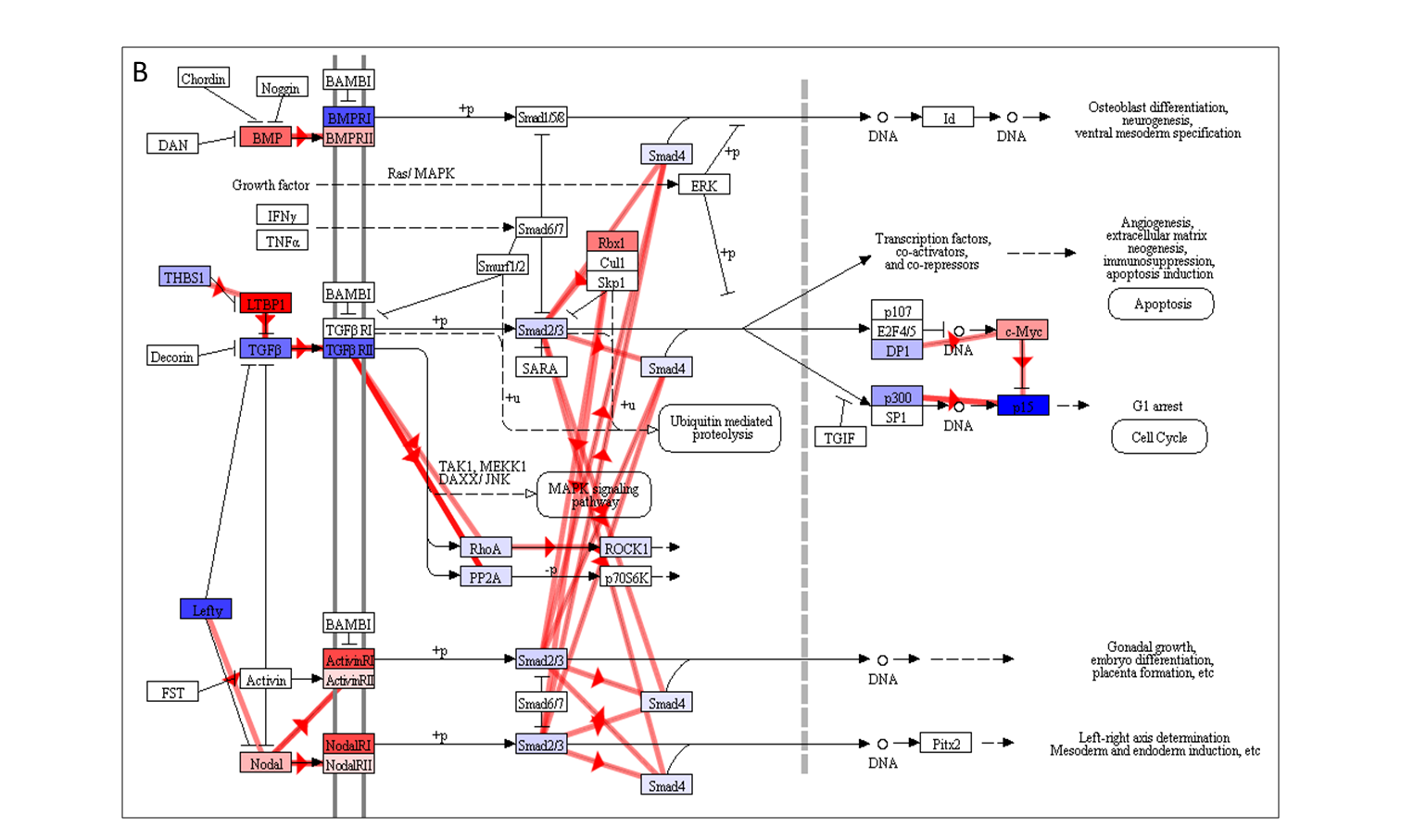

Supplement: Supplementary file 1 [file jpm-09-00034-s001.zip › Supplementary Figure S1B.TIF]

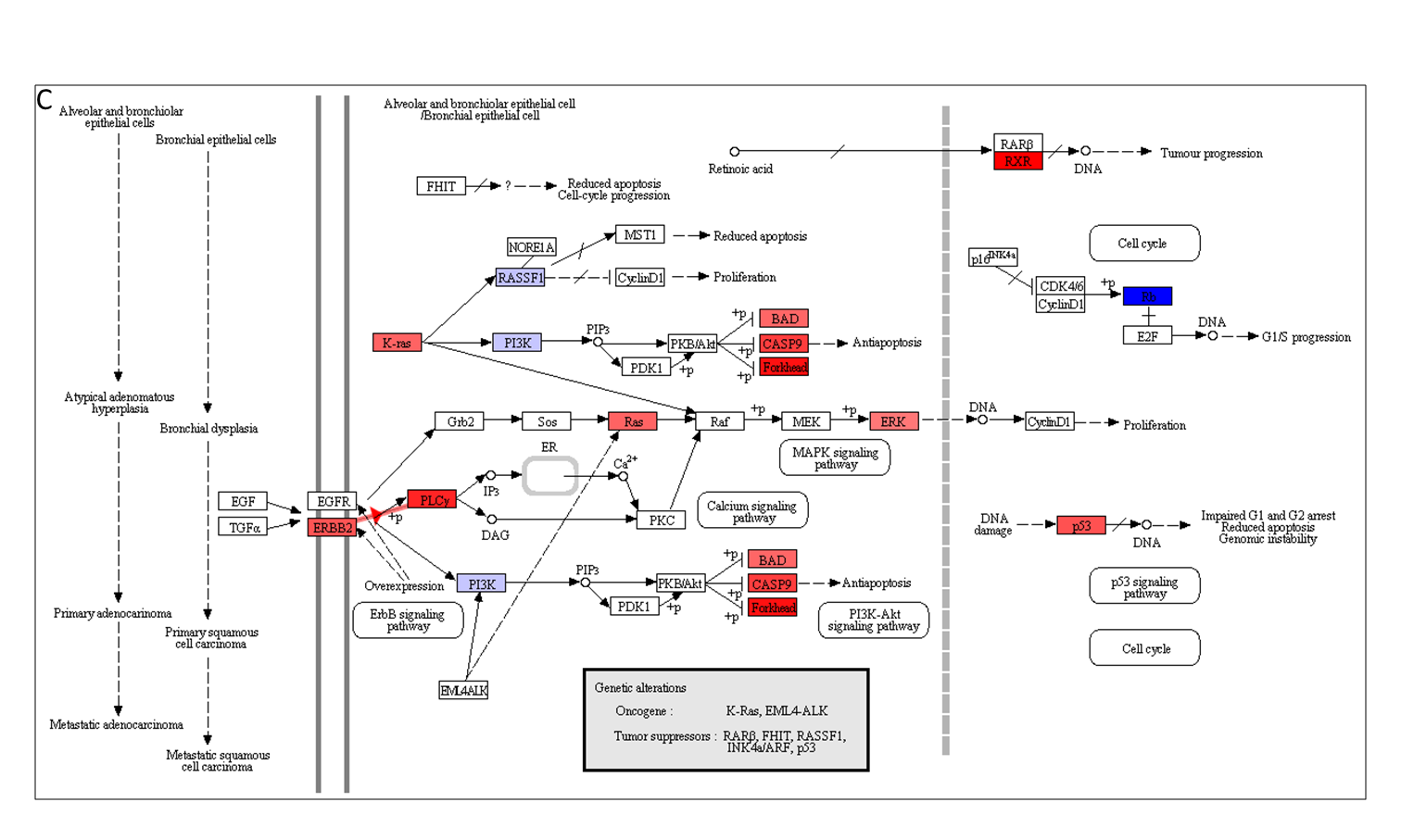

Supplement: Supplementary file 1 [file jpm-09-00034-s001.zip › Supplementary Figure S1C.TIF]

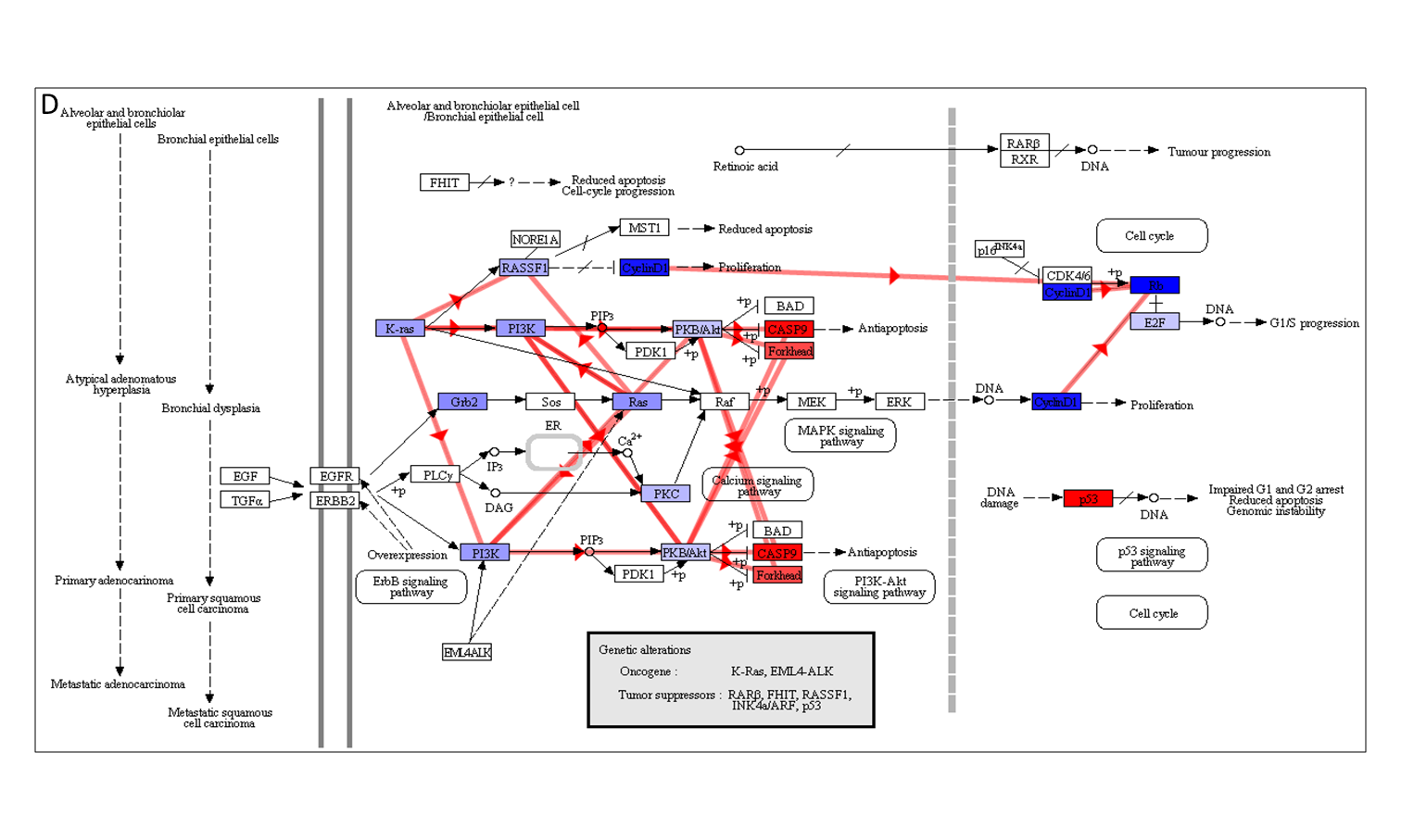

Supplement: Supplementary file 1 [file jpm-09-00034-s001.zip › Supplementary Figure S1D.TIF]
